# Simplified antibiotic regimens for treating neonates and young infants with severe infections in the Democratic Republic of Congo: a comparative efficacy trial

**DOI:** 10.1186/s40748-018-0076-2

**Published:** 2018-04-18

**Authors:** Adrien Lokangaka, Melissa Bauserman, Yves Coppieters, Cyril Engmann, Shamim Qazi, Antoinette Tshefu, Carl Bose

**Affiliations:** 1 Faculté de Médecine, Université de Kinshasa, Kinshasa School of Public Health, PO Box 11850, Kinshasa/Lemba, Democratic Republic of Congo; 2 University of North Carolina at Chapel Hill, Chapel Hill, NC USA; 3 School of Public Health, Université libre de Belgique (ULB), Brussels, Belgium; 4 University of Washington, Seattle, Washington USA; 5 World Health Organization, Geneva, Switzerland

**Keywords:** Neonatal infection, Simplified antibiotic regimen, Community-based treatment

## Abstract

**Background:**

One-quarter of neonatal and infant deaths are due to infection, and the majority of these deaths occur in developing countries. Standard treatment for infection, which includes parenteral treatment only, is often not available in low-resource settings. Infant mortality will not be reduced in developing countries without a reduction in deaths due to infection. We participated in a multi-site trial that demonstrated the effectiveness of three simplified antibiotic regimens compared to standard treatment (The AFRINEST Trial: parent study). For this report, we examined the site-specific data for the Democratic Republic Congo (DRC), the most impoverished of the countries that participated in the study, to determine if outcomes in the DRC were similar to outcomes across all sites.

**Methods:**

The parent study was an individually randomized, open-label, equivalence trial. Infants with clinical signs of severe infection were randomized to receive one of four regimens: 1) injectable penicillin-gentamicin for 7 days (standard therapy; regimen A), 2) injectable gentamicin and oral amoxicillin for 7 days (regimen B), 3) injectable penicillin-gentamicin for 2 days then oral amoxicillin for 5 days (regimen C), or 4) injectable gentamicin for 2 days and oral amoxicillin for 5 days (regimen D). In the DRC, we enrolled 574 infants, of whom 560 met the per-protocol criteria for analysis of treatment effect. The main outcome was treatment failure within the first week of enrollment.

**Results:**

Treatment failure occurred in 52 (9.3%) infants: 17 (11.6%) with the referent treatment regimen, 13 (9.6%) with regimen B (risk difference [RD] -2.0%; CI -9.2% to 5.2%), 13 (9.0%) with regimen C (RD -2.6%; CI -9.6% to 4.4%), and 9 (6.7%) with regimen D (RD -5.0%; CI -11.7% to 1.7%).

**Conclusion:**

As in the parent study, the risk difference between each of the experimental treatments and the reference treatment suggests equivalence. These findings suggest that the conclusion from the parent study, that a simplified antibiotic regimen can be used for the community-based management of possible severe infection in young infants where referral to a hospital for standard care is often not possible, is true in the DRC. We speculate that the widespread use of a simplified, community-based treatment could result in increased coverage with treatment and improved survival in poor areas.

**Trial registration:**

ACTRN12610000286044 on April 9, 2010.

## Background

Reducing child mortality continues to be one of the most vexing challenges in this Sustainable Development Goals era. An estimated 5.6 million children died in 2016; approximately 2.6 million of these deaths occurred in the neonatal period [1]. Of these child deaths, the proportion occurring in neonates and young infants (infants 0–2 months) continues to rise [2]. Evidence-based research, policies, programs and advocacy that target this age group are urgently needed to combat this problem.

Infections are among the leading causes of neonatal and young infant mortality [1]. In 2012, over 0.66 million infants died of serious bacterial infections, such as pneumonia, sepsis, and meningitis [3]. Until 2015, the World Health Organization (WHO) recommended that all neonates and young infants with possible serious bacterial infections (PSBI) be treated in hospitals with injectable antibiotic therapy for 7–10 days [4]. However, this recommendation is difficult to implement in many low-income countries, particularly in rural areas. The Democratic Republic of Congo (DRC), located in central Africa and the fourth most populous African country, is particularly vulnerable to the challenges of implementing these WHO recommendations. In the DRC, nearly all health care in rural areas is provided through health centers that do not typically provide inpatient care. Providers in health centers may refer some patients to their area hospital if inpatient care is advisable and feasible; however, distances from health centers to hospitals vary widely, ranging from less than one mile to 60 miles, the terrain is challenging and obtaining transport poses immense difficulties. For the patient who makes it to a referral hospital, the hospitals are lacking in sufficiently trained health care providers, and equipment and essential medicines are often absent [5]. As a result of these barriers, many infants with PSBI are not taken to hospitals, and if they do get there are either untreated or inadequately treated. These barriers and inadequacies of treatment contribute to the neonatal mortality in these regions.

Because of these barriers to recommended treatment in the DRC and other resource-limited countries, a collaboration of investigators from the WHO and the Universities of Kinshasa and North Carolina participated in a multi-site study (five sites from three countries) that examined the efficacy of four simplified regimens of outpatient antibiotic therapy for the treatment of neonates and young infants with PBSI [6]. The results of the multi-center study (the African Neonatal Sepsis Trial: AFRINEST Trial) were published in the *Lancet* and suggest that these infections could be treated effectively outside of referral hospitals, in health centers or homes [7]. The WHO modified its recommendations for treatment of PSBI based, in part, on the results of this study [8]. However, each of the five sites had unique demography, geography and healthcare infrastructure that might predict variation in efficacy among sites. The objective of this report is to examine the DRC site-specific data from the multi-site study to determine the comparative efficacy of these treatment regimens in the cohort enrolled in the DRC, the most rural and impoverished of the study sites.

## Methods

### Study site

Our site was in rural areas of the North and South Ubangi districts in the province of Equateur in northern DRC. The overall population of the study area was roughly 400,000. We included 30 health areas, each served by a health center. Health centers are the primary level facilities staffed by one trained nurse who provides treatment to ill infants.

#### Study design:

The parent study was a multi-site, individually randomized, open-label, equivalence trial [6].

A. ***Eligibility Criteria***

Young infants and neonates (0–59 days old) with signs of PSBI and whose families did not accept or could not access inpatient hospital care for whatever reason were enrolled and randomized. Signs of PSBI included: not feeding well, movement only when stimulated, severe chest indrawing and axillary temperature > 38.0 °C or < 35.5 °C. We excluded infants who had very low weight (< 1500 g) at the time of presentation, had been hospitalized for illness in the previous two weeks or prior to inclusion in the study, any sign of critical illness (unconscious, convulsions, unable to feed at all, apnea, unable to cry, cyanosis, dehydration, bulging fontanel), major congenital malformations inhibiting oral antibiotic intake, active bleeding requiring transfusion, surgical conditions needing hospital referral, and persistent vomiting (vomiting following three attempts to feed the baby within one-half hour).

B. ***Surveillance***

We developed an active surveillance system in order to maintain a registry of infants in the study communities. At the beginning of the trial, we used community health workers (CHWs) to conduct a household census in order to identify all births and pregnant women. CHWs repeated the household census every three to four months. We incorporated other methods to discover pregnancies and births: self-reporting of pregnancies to a CHW, identification of pregnant women at antenatal clinics in the community health facilities, and referrals from traditional birth attendants (TBAs) or other key informants.

C. ***Enrollment***

CHWs visited the homes of newborns on postnatal days 1, 3, 7, 14, 21, 28, 35, 42, 49 and 60. During these home visits, the CHWs provided standardized advice to the family regarding newborn care, as described in the WHO/UNICEF Joint Statement on home-based care of newborns [6]. At each home visit, CHWs assessed the newborn for signs of illness and counseled the families on recognition of danger signs of infection. Young infants who exhibited danger signs were referred to a health center for evaluation.

All infants who presented with danger signs were evaluated by a study nurse. This assessment was in addition to and independent of an assessment performed by a health center provider, and occurred either in the health center or in the home. If the study nurse confirmed that a danger sign and PSBI was present, the infant was referred to local hospital facility, as recommended in the WHO Integrated Management of Children Illness (IMCI) guidelines [9]. If the family refused to accept hospital referral despite the best efforts of the study nurse, they were considered for enrollment in the study.

D. **Consent**

Consent for study participation was obtained by the study nurse at the health facility or at home in the presence of a witness. Consent included detailed oral communication about the trial and study procedure in the study participant’s native language. Illiterate parents were asked to provide a thumbprint on the consent form; literate parents were requested to sign the consent form.

E. ***Randomization and Allocation Concealment***

Prior to randomization, infants with PSBI were stratified by age at presentation (< 7 days old and 7 to 59 days old) and assigned to one of four treatment regimens. For allocation concealment, the treatment code for each study infant was sealed in an envelope, one color for each age stratum. Each cluster (a group of health centers) was given envelopes for a set of blocks. When the first infant was enrolled in a cluster in a stratum, the first envelope of the first block for that age stratum was opened, and the infant was treated according to the treatment code inside. When the next infant was enrolled, the next envelope of the block was opened.

F. ***Treatment Regimens***

Each patient was randomized to one of four treatment regimens:

- Treatment regimen A (reference treatment): gentamicin (desired range 4–5 mg/kg/day) by intramuscular (IM) injection once daily, and procaine penicillin (desired range 40,000–50,000 units/kg/day) by IM injection once daily for 7 days (14 injections in total)
- Treatment regimen B: gentamicin (desired range 4–5 mg/kg/day) by IM injection once daily and oral amoxicillin (desired range 75–100 mg/kg/day) twice daily for 7 days (7 injections in total)
- Treatment regimen C: gentamicin (desired range 4–5 mg/kg/day) by IM injection once daily and procaine penicillin (desired range 40,000–50,000 units/kg/day by IM injection once daily for 2 days; thereafter oral amoxicillin (desired range 75–100 mg/kg/day) for 5 days (4 injections in total)
- Treatment regimen D: gentamicin (desired range 4–5 mg/kg/day) by IM injection once daily for two days and oral amoxicillin (desired range 75–100 mg/kg/day) twice daily for 7 days; (2 injections in total)

### Study outcomes

Treatment failure within day 1–8 following enrollment was the primary outcome and was defined as any one of the following: death, clinical deterioration (hospitalization, emergence of any sign of critical illness, a new sign of severe infection, or re-emergence of a sign of severe infection on day 4 after it had initially disappeared), no improvement in clinical condition by day 4 (if single sign of severe infection was present at enrollment, persistence of the sign, and if multiple signs were present at enrollment, persistence of > 1 sign), no clinical cure by day 8 (persistence of any sign of severe infection on day 8), development of a serious adverse effect to the study antibiotics, or withdrawal of informed consent, any time between days 1–8.

### Sample size and analysis plan

The sample size for the parent study was based on an estimated incidence of severe bacterial infection among neonates and young infants of 5%. The analytic plan was to compare failure rates of treatment regimens B, C and D to failure rates following treatment regimen A. Comparisons were made for similarity of effectiveness defined by the upper limit of the 95% confidence interval of the differences in failure rate lying below the similarity margin of + 5%. In the parent study, the required sample for 90% power to demonstrate the similarity of any two treatments assuming that the true failure rates with the reference treatment and the experimental treatment regimens were identical (assumed to be 10%) was estimated to be a total 3040. An additional 560 infants were added to the planned enrollment to allow for failures of adherence to the protocol to allow for both per-protocol and intention-to-treat analyses. Sample size calculations for individual sites were not calculated because there was no a priori intent to perform sit-specific analyses. Therefore, the sample size for the DRC was the number enrolled at this site during the duration of the parent study.

We conducted these single site analyses using STATA version 12.0 (StataCorp, College Station, TX). We analyzed the primary outcome (treatment failure) per-protocol, which is considered a more conservative analysis than intention to treat (ITT) analysis for equivalence studies. We evaluated the difference in the risk of treatment failure between the reference treatment (regimen A) and all other treatments together with a 95% confidence interval. We report planned investigation of secondary outcomes and comparisons between features of the DRC and the parent study cohort using descriptive statistics only.

## Results

**Fig. 1:**
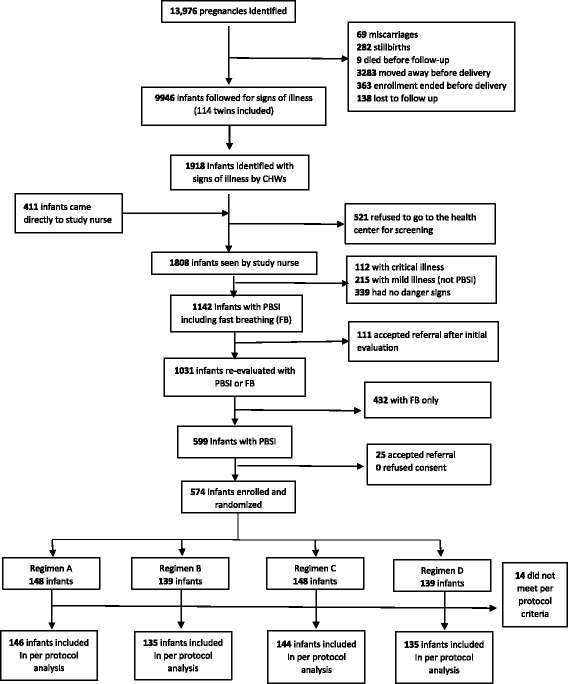
CONSORT Diagram of Democratic Republic of Congo study cohort. Origin of the study cohort in the Democratic Republic of Congo. The diagram illustrates the origin of the 560 infants whose outcomes were analyzed for treatment effect. Boxes to the right indicate when and why infants were excluded

From September 17, 2012 to June 28, 2013, we enrolled 574 infants (Fig. 1), 16% of the 3564 infants enrolled in the parent study. Mean maternal age at enrollment was 25 years, and 47% had no formal education. Nearly all (95.3%) attended at least one antenatal clinic visit. Among all infants, 398 (69%) were born in health centers, 66 (12%) in hospitals, and 85 (15%) at home. Compared to all participants in the parent study, participants from the DRC had lower maternal education and more used solid fuel for cooking (Table 1).

**Table 1:** Demographics for DRC Site compared to AFRINEST study population

	DRC Site	AFRINEST Study Population
	*N* = 574	*n* = 3564
Maternal age (years)		
Mean (SD)	25.0 (6.4)	25.9 (5.8)
< 20 years	122 (21%)	440 (12%)
≥ 20 years	385 (67%)	1969 (84%)
Not known	67 (12%)	110 (3%)
Maternal education (years)		
No formal school attendance	271 (47%)	625 (18%)
< 12	292 (51%)	2025 (57%)
≥ 12	11 (2%)	896 (25%)
unknown	0 (0%)	10 (< 1%)
Cooking place and fuel (n)		
Indoor with solid fuel	315 (55%)	1534 (43%)
Outdoor with solid fuel	259 (45%)	771 (22%)
No solid fuel	0	1279 (36%)
Had at least 1 antenatal care visit (n)		
Yes	555 (97%)	3371 (95%)
No	19 (3%)	184 (5%)
Not known	0	9 (< 1%)
Number of previous live births		
1	145 (25%)	847 (24%)
2–3	231 (40%)	1377 (39%)
> 4	198 (35%)	1342 (38%)
Not known	0	8 (< 1%)

In the DRC, the randomization process allocated 148 (25.2%) infants to treatment regimen A, 139 (24.2%) to regimen B, 148 (25.2%) to regimen C, and 139 (24.2%) to regimen D. Enrollment occurred soon after birth for many infants; 198 (34%) were enrolled in their first week of life. Mothers and infants in each group had similar baseline characteristics (Table 2). At enrollment, the most common presenting signs was fever (57.0%). We enrolled 75 infants (13.1%) with two or more signs.

**Table 2:** Baseline characteristics of enrolled infants

Regimen^a^		*A*	*B*	*C*	*D*
Number of infants enrolled		148	139	148	139
Age at enrollment (days)	Mean (SD)	17 (15)	17 (15)	17 (16)	20 (17)
	< 7 days	54 (36.5%)	46 (33.1%)	55 (37.2%)	43 (30.9%)
	≥ 7 days	94 (63.5%)	93 (66.9%)	93 (62.8%)	96 (69.1%)
Sex	Male	75 (50.7%)	76 (54.7%)	70 (47.3%)	81 (58.3%)
Respiratory rate	Mean (SD)	69 (19)	69 (19)	70 (20)	67 (19)
	< 60	54 (36.5%)	47 (33.8%)	52 (35.1%)	57 (41.0%)
	60–70	27 (18.2%)	21 (15.1%)	26 (17.6%)	16 (11.5%)
	70–79	22 (14.9%)	33 (23.7%)	26 (17.6%)	32 (23.7%)
	80–89	24 (16.2%)	20 (14.4%)	25 (16.9%)	19 (13.7%)
	90–99	8 (5.4%)	9 (6.5%)	9 (6.1%)	8 (5.8%)
	≥ 100	13 (8.8%)	9 (6.5%)	10 (6.8%)	7 (5.0%)
Temperature	< 35.5	6 (4.1%)	8 (5.8%)	12 (8.1%)	12 (8.6%)
	35.5–37.9	39 (26.4%)	47 (33.8%)	44 (29.7%)	33 (23.7%)
	≥38.0–38.9	96 (64.9%)	70 (50.4%)	80 (54.1%)	76 (54.7%)
	≥39.0	7 (4.7%)	14 (10.1%)	12 (8.1%)	18 (12.9%)
Poor feeding		23 (15.5%)	22 (15.8%)	26 (17.6%)	25 (18.0%)
Movement only on stimulation		3 (2.0%)	5 (3.6%)	3 (2.0%)	1 (0.7%)
Severe chest indrawing		33 (22.3%)	40 (28.8%)	32 (21.6%)	31 (22.3%)
Number of signs at enrollment	1	129 (87.2%)	120 (86.3%)	128 (86.5%)	120 (86.3%)
	2	18 (12.2%)	18 (12.9%)	17 (11.5%)	16 (11.5%)
	≥ 3	1 (0.7%)	1 (0.7%)	1 (0.7%)	3 (2.2%)

^a^Regimen A: IM Gentamicin and IM Penicillin × 7 days; Regimen B: IM Gentamicin and oral amoxicillin × 7 days; Regimen C: IM Gentamicin × 7 days and IM Penicillin × 2 days then oral amoxicillin × 5 days; Regimen D: IM Gentamicin × 2 days and oral amoxicillin × 7 days

Almost all infants, 98% received all treatment doses as per-protocol analysis, and 97% received all independent outcome assessment visits (Table 3). We excluded 14 infants from our analysis of treatment effect because they did not receive all treatment doses and adequate follow-up as required by the study protocol [6].

**Table 3:** Treatment adherence and follow-up of enrolled infants

Regimen^a^	*A*	*B*	*C*	*D*
Number of infants enrolled	148	139	148	139
Treatment adherence				
Received all treatment doses as per-protocol	146 (98.6%)	137 (98.6%)	144 (97.3%)	135 (97.1%)
Did not receive all doses, but met per-protocol analysis criteria	2 (1.4%)	1 (0.7%)	4 (2.7%)	4 (2.9%)
Did not meet per-protocol analysis criteria for treatment	0	1^b^ (0.7%)	0	0
Follow up by independent outcome assessor				
Received all independent outcome assessment visits	145 (98.0%)	133 (95.7%)	145 (98.0%)	133 (95.7%)
Did not receive all independent outcome assessment visits, but met per-protocol analysis criteria	2 (1.4%)	3 (2.2%)	2 (1.4%)	4 (2.9%)
Did not meet per-protocol analysis criteria for assessment	2 (1.3%)	4 (3.0%)	4 (2.7%)	4 (3.0%)
Included in per-protocol analysis (met both treatment and assessment criteria)	146 (98.6%)	135 (97.1%)	144 (97.3%)	135 (97.1%)

^a^Regimen A: IM Gentamicin and IM Penicillin × 7 days; Regimen B: IM Gentamicin and oral amoxicillin × 7 days; Regimen C: IM Gentamicin × 7 days and IM Penicillin × 2 days then oral amoxicillin × 5 days; Regimen D: IM Gentamicin × 2 days and oral amoxicillin × 7 days

^b^Infant failed to meet both per-protocol treatment adherence and outcome assessment criteria

Treatment failure occurred in 52 (9.2%) infants (Table 4). When compared to the reference treatment, the risk difference with regimen B was − 2.0% (95% CI: -9.2 to 5.2), with regimen C: − 2.6% (− 9.6 to 4.4), and with regimen D -5.0% (− 11.7 to 1.7) (Table 3). Among treatment failures, 11 infants died; 9 had the appearance of a sign of critical illness; 5 had a new sign of serious infection; and 21 had no improvement in clinical condition by day 4. Treatment failure occurred most commonly on day 4 following enrollment.

**Table 4:** Primary and secondary outcomes in enrolled infants–per-protocol analysis

Regimen^a^	*A*	*B*	*C*	*D*
Number of infants analyzed	146	135	144	135
Treatment failure n (%)	17 (11.6%)	13 (9.6%)	13 (9.0%)	9 (6.7%)
Risk difference % (95% CI)	referent	-2.0 (− 9.2 to 5.2)	-2.6 (−9.6 to 4.4)	-5.0 (−11.7 to 1.7)
Reason for treatment failure				
Death	1 (0.7%)	2 (1.5%)	6 (4.2%)	2 (1.5%)
Appearance of a sign of critical illness	2 (1.4%)	2 (1.5%)	2 (1.4%)	3 (2.2%)
Appearance of a new sign of serious infection	1 (0.7%)	2 (1.5%)	2 (1.4%)	0
SAE other than death	0	0	0	0
Hospitalization	0	0	0	0
No improvement in clinical condition by day 4	10 (6.8%)	6 (4.4%)	1 (0.7%)	4 (3.0%)
Reappearance of inclusion sign between days 5–8	2 (1.4%)	1 (0.7%)	2 (1.4%)	0
Presence of inclusion sign on day 8	1 (0.7%)	0	0	0

^a^Regimen A: IM Gentamicin and IM Penicillin × 7 days; Regimen B: IM Gentamicin and oral amoxicillin × 7 days; Regimen C: IM Gentamicin × 7 days and IM Penicillin × 2 days then oral amoxicillin × 5 days; Regimen D: IM Gentamicin × 2 days and oral amoxicillin × 7 days

## Discussion

The AFRINEST Trial, a multi-national study, investigated the safety and effectiveness of simplified regimens for the management of possible serious bacterial infection among infants in resource-poor community settings. This study enrolled 3564 infants in five sites in three countries (Kenya, DRC, and Nigeria) [7]. In the parent study, four week-long treatment regimens were compared. The outcomes of infants treated with three regimens of antibiotics that included combinations of parenteral (intramuscular) and oral antibiotics were compared to outcomes in a reference group treated with daily doses of parenteral antibiotics, the standard care. Treatment failure occurred in 6.8% of infants, but the risk differences between the experimental treatment regimens and the reference treatment were within the pre-specified 5% similarity margin. The conclusion from this study was that treatment with these regimens was equivalent to the standard care.

The purpose of the study reported in this manuscript was to determine whether the results from this multi-national study could be reasonably extrapolated to the DRC. The DRC is a unique environment compared to the other sites for several reasons. First, mothers had less education compared to other sites. Second, the socio-economic status is lower in the DRC. Despite the abundant natural resources of the country, the population of the DRC is among the poorest in the world [10, 11]. According to the 2013 Human Development Report, the DRC ranks last (186th) with a poverty ratio of about 80% [12]. This compares to ranks of 153rd and 145th for Nigeria and Kenya. Third, the DRC has high fertility rates and bigger families. The total fertility rate in the DRC is 6.6, [13] compared to 5.5 in Nigeria [14] and 3.9 in Kenya [15].

As a participant in the multi-national study, we examined the safety and efficacy of simplified antibiotic regimens compared with the reference treatment for the management of neonates and young infants with PSBI among infants enrolled in the study. Our site-specific data demonstrated similarity between each of the experimental treatment regimens and the reference treatment. Treatment failure varied among groups from 11.6% to 6.7%. Treatment regimen D, which had only two injections of gentamicin, had the smallest proportion of treatment failure. The risk difference in treatment among the three simplified regimens and the reference treatment varied from − 2.0% to − 4.9%. The upper limits of the confidence intervals for all risk differences were less than the pre-specified limit that defined similarity (5%) with the exception of regimen B in which the upper limit of the confidence interval was 5.2%. As expected, the confidence intervals were greater in our smaller tudy population compared to the larger population in the multi-national study. However, it was encouraging to observe that, although the risk of treatment failure for all groups was greater than in the larger study population, all risk differences compared to the referent arm were negative.

Most treatment failures occurred on day 4, and the most common reason for treatment failure was the persistence of the danger signs on day 4. Collectively, these findings are similar to those observed in the multi-national study. In view of these collective similarities with the multi-national, it can reasonably be inferred that treatment regimens for young infants with signs of serious bacterial infection tested in the multi-national study would be equally effective in the DRC.

In response to the publication of the parent study, and a companion study investigating the effectiveness of a simplified antibiotic treatment regimen for fast breathing [16, 17], several respondents expressed concerns about the context, observations and inferences drawn from these studies [18–20]. These concerns included the unexpectedly low mortality rates in all arms of the study, the use of criteria for PSBI that have high sensitivity but low specificity, the inherent over-treatment of infants with viral rather than bacterial infection, and the potential for emergence of resistance to these antibiotics resulting from the widespread adoption of the simplified regimens. These concerns were addressed in a response by investigators from the parent study [21]. We can only add that the parent study was a pragmatic trial to determine if a simplified alternative to the recommended treatment for PBSI, that is not available to most infants in low resource environments, would be equally effective. Despite the limitations imposed by the study design, the WHO concluded that the evidence was sufficiently compelling to recommend the simplified regimens in low resource settings when the standard treatment is not available [8]. Our site-specific analyses suggest that these recommendations should be adopted in the DRC, and perhaps other similarly impoverished and very low resource environments.

### Strengths and limitations

This is the first study in the DRC that compared simplified treatment regimens utilizing oral antibiotic regimens in an outpatient setting for the management of neonatal SBIs. This study was conducted within the context of the existing health structure. However, the protocol was highly supervised; eligibility was confirmed by specially trained study nurses and assessment visits were conducted by the most qualified nurses among them who were not part of the clinical care team. This level of oversight was necessary to ensure the quality of the research but would not typically be available during standard clinical care. Therefore, although the study methodology could be used as a model for capacity-building of the existing health system, scale up might require additional resources. Absent these resources, it is possible that outcomes of simplified treatments might not have compared as favorably to standard treatment. With the support of the WHO, we are currently investigating the feasibility of scale up of simplified regimens in another low-resource area of the DRC. This ongoing study will help address concerns about feasibility. The study was conducted in one of the poorest regions of the country. The confirmation of effectiveness in this area suggests that the effectiveness of these simplified treatments can be generalized to more affluent areas of the country, where scale up might be more feasible.

Although the multi-national study had sufficient statistical power to demonstrate equivalence between treatments, it was not powered for site-specific outcomes. Therefore, our results should be interpreted with some caution. In addition, the relatively low mortality rate among all treatment groups may reflect the intense surveillance of the population. This close surveillance may have resulted in earlier identification of high-risk infants and earlier referral for health care. Later identification might have occurred in the absence of the study resulting in more severe illness at the initiation of antibiotic treatment, and less effectiveness of simplified treatment regimens and higher mortality.

### Implications of the results

Community-based treatments are more practical because they do not require inpatient care that is not available to many children in rural areas of the DRC. The most simplified treatment regimen may be particularly useful because it includes primarily on oral treatment. We speculate that the widespread use of this strategy for treating neonates and young infants with PSBI would result in more infants treated more effectively. This, in turn, would reduce mortality among young infants.

## Conclusion

Simplified antibiotic regimens for treating infants in rural DRC with PSBI appear to be acceptable, feasible, safe, and effective. Since the most simplified regimen using mainly oral antibiotic and only two injections proved as effective as the WHO-recommended treatment, scaling up this regimen will more likely result in more infants treated effectively and result in reduced mortality in poor areas where hospital care is costly and inaccessible.

## Abbreviations

CHW: community health worker
DRC: Democratic Republic of Congo
IMCI: Integrated Management of Child Illness
ITT: Intention to Treat
PSBI: possible serious bacterial infection
TBA: Traditional Birth Attendant
WHO: World Health Organization
